# Prostate cancer incidence and mortality linked to metalworking fluid exposure: a systematic review and meta-analysis

**DOI:** 10.3389/fonc.2024.1491159

**Published:** 2025-01-30

**Authors:** Zahra Moradpour, Amin Barik, Goljamal Jorjani, Mohammad Reza Taherian, Sepideh Tousizadeh, Aram Halimi, Yaser Soleimani, Mobina Karimian, Tina Khavari, Fateme Azizi Kalankari, Fatemeh Asadipour, Mojtaba Azari, Niloofar Yousefzadeh Shakouri, Saeideh Karamian, Nasser Bahari, Alireza Mosavi Jarrahi

**Affiliations:** ^1^ Department of Occupational Health Engineering, Environmental Health Research Center, Golestan University of Medical Sciences, Gorgan, Iran; ^2^ Department of Health, Safety and Environment, School of Public Health and Safety, Shahid Beheshti University of Medical Sciences, Tehran, Iran; ^3^ Department of Epidemiology, School of Public Health, Shahid Beheshti University of Medical Sciences, Tehran, Iran; ^4^ Student Research Committee, School of Public Health, Shahid Beheshti University of Medical Sciences, Tehran, Iran; ^5^ Student Research Committee, Shahrekord University of Medical Sciences, Shahrekord, Iran; ^6^ Department of Environmental Health Engineering, School of Health, Shahrekord University of Medical Sciences, Shahrekord, Iran; ^7^ Research Center for Social Determinants of Health, Research Institute for Metabolic and Obesity Disorders, Research Institute for Endocrine Sciences, Shahid Beheshti University of Medical Sciences, Tehran, Iran; ^8^ Medical School, Shahid Beheshti University of Medical Sciences, Tehran, Iran; ^9^ Department of Occupational Health Engineering, Student Research Committee, Sabzevar University of Medical Sciences, Sabzevar, Iran; ^10^ Student Research Committee, Tabriz University of Medical Sciences, Tabriz, Iran; ^11^ Department of Social Medicine, School of Public Health and Safety, Shahid Beheshti University of Medical Sciences, Tehran, Iran

**Keywords:** metalworking fluids, prostate cancer, SMR, RR, mortality, morbidity, meta-analysis

## Abstract

**Background:**

Prostate cancer is the second most diagnosed cancer in men globally, with high prevalence in North America, Europe, and Australia. Occupational exposures, including metalworking fluids (MWFs), have emerged as a potential risk factor for prostate cancer, yet comprehensive studies on this association are limited.

**Objective:**

This study aims to systematically review and conduct a meta-analysis to examine the incidence and mortality of prostate cancer linked to MWF exposure.

**Methods:**

A systematic review and meta-analysis were conducted following the PRISMA guidelines. A comprehensive search strategy was developed to identify relevant studies from PubMed, Scopus, Embase, and Web of Science. Inclusion criteria encompassed studies reporting on the association between MWF exposure and prostate cancer incidence or mortality. Data extraction and risk of bias assessment were performed independently by two reviewers, with discrepancies resolved by a third reviewer. Statistical analyses were conducted using STATA version 17.

**Results:**

The search identified 1376 unique references, with 5 studies meeting the inclusion criteria for the meta-analysis. These studies, conducted in the USA, primarily involved auto workers and reported Standardized Mortality Ratio (SMR), Relative Risk (RR) and 95% Confidence Interval (95%CI) measures. Meta-analysis revealed an overall RR of 1.06 (95% CI: 1.01-1.11) for prostate cancer incidence and an overall SMR of 1.20 (95% CI: 1.09-1.31) for prostate cancer mortality, indicating a statistically significant increased risk and mortality among MWF-exposed workers.

**Conclusions:**

These findings carry significant implications for workplace safety regulations. Given the observed association between MWF exposure and prostate cancer risk, it is imperative to minimize occupational exposure through the implementation of effective engineering controls, personal protective equipment, and substitution of less hazardous fluids. Regular health surveillance and education programs for workers in industries utilizing MWFs are also essential to mitigate risk. Additionally, regulatory agencies should consider revising exposure limits and safety guidelines to account for emerging evidence on the carcinogenic potential of newer MWF formulations.

## Introduction

Prostate cancer is the second most diagnosed cancer in men globally. Prostate cancer’s prevalence varies across regions and is intricately linked to demographic, genetic, and lifestyle factors. As the second most common cancer among men globally, its impact is particularly notable in North America, Europe, and Australia, where higher incidence rates are observed. The rise in prostate cancer cases is closely associated with age, with the majority of diagnoses occurring in men over 50. Ethnic disparities add another layer of complexity, with African American men experiencing higher incidence rates compared to their European or Asian counterparts (1–5).

The advent of screening tools, particularly the prostate-specific antigen (PSA) test, has significantly contributed to the early detection of prostate cancer. While early diagnosis allows for timely intervention and improved outcomes, the debate surrounding the benefits and risks of widespread screening persists. The age-old adage “knowledge is power” holds true in the context of prostate cancer, where informed decisions about screening and treatment can influence individual health trajectories (6–9).

Prostate cancer generally boasts high survival rates, particularly when detected at an early, localized stage. The favorable prognosis is further enhanced by advancements in treatment modalities, ranging from active surveillance for low-risk cases to surgery, radiation therapy, and hormonal therapies for more advanced disease. Yet, the landscape of prostate cancer treatment is evolving, with ongoing research exploring novel therapeutic approaches and personalized medicine strategies (10–12).

Beyond geographical variations, the prevalence of prostate cancer is significantly influenced by genetic and ethnic factors. While the reasons for the higher incidence rates among African American men remain under investigation, it underscores the importance of considering genetic and ancestral determinants in understanding cancer risk. Genetic susceptibility, coupled with environmental exposures, contributes to the complex etiology of prostate cancer, making it a multifaceted disease with variable outcomes (13–20).

In recent years, the nexus between occupational exposures and cancer risk has become an essential facet of cancer research. Among the myriad occupational hazards, MWFs have emerged as agents warranting careful consideration due to their ubiquitous use in manufacturing and machining processes. These fluids, comprising a complex mixture of oils, emulsifiers, anti-corrosion agents, and biocides, are integral to metalworking operations. However, the potential health implications of prolonged exposure to MWFs, particularly in the context of prostate cancer, remain a focal point of investigation (21–26).

MWFs, integral to manufacturing and machining processes, are broadly classified into three main types based on their composition. The first type, Straight Oils, consists of mineral oils without water content, recognized for their exceptional lubrication and cooling properties. Although these fluids are less likely to cause skin irritation, they may lead to dermatitis in some cases. Soluble Oils, the second category, are mixtures of mineral oils and water, forming emulsions. These fluids offer good cooling properties, are cost-effective, but carry the potential for skin irritation. The presence of water in soluble oils may also facilitate the growth of bacteria and fungi. The third type, Synthetic or Semi-Synthetic Fluids, is formulated with water-soluble synthetic compounds or a combination of synthetic and mineral oils. Designed to enhance performance and reduce environmental impact, these fluids present a lower risk of skin irritation when compared to straight oils and soluble oils. Understanding these distinctions is crucial for assessing the potential health impacts of Metalworking Fluids, as variations in chemical composition may influence their carcinogenic potential and biological interactions. Understanding the distinctions among these MWF types is crucial for assessing their potential health impacts, as the chemical composition may vary, influencing their carcinogenic potential and biological interactions (22, 24, 26–32).

While the prevalence of prostate cancer and the potential role of occupational exposures have been subjects of extensive research, a comprehensive synthesis of the literature specifically focusing on prostate cancer patients exposed to MWFs is notably absent. This research undertakes a systematic review and meta-analysis to bridge this critical gap, offering a holistic examination of the SMR in individuals diagnosed with prostate cancer and with a history of exposure to MWFs. The rationale for this investigation is rooted in the urgency to unravel the nuanced relationship between occupational exposures and cancer outcomes, particularly in a cohort where MWF exposure may act as a potential contributory factor.

## Methods

A comprehensive detail of the protocol of this study has been already published (33).This study was conducted following the Preferred Reporting Items for Systematic Reviews and Meta-Analyses (PRISMA) guidance (34).

### Search strategy

A comprehensive search strategy was developed to identify relevant studies in electronic databases. The search strategy was implemented in four major databases: PubMed, Scopus, Embase, and Web of Science. The search queries were designed to capture studies related to metalworking fluids and prostate cancer. **Table 1** outlines the specific search queries used in each database. This search was conducted until 21/12/2024.

**Table 1:** Search strategy in databases.

Databases	Search Query
PubMed	((“metalworking fluid*”[Title/Abstract] OR “metal working fluid*”[Title/Abstract] OR (“metalworking”[Title/Abstract] AND fluid*[Title/Abstract]) OR “cutting fluid*”[Title/Abstract] OR “metal fluid*”[Title/Abstract] OR “MWF”[Title/Abstract] OR “MWFs”[Title/Abstract] OR “lubricant fluid*”[Title/Abstract] OR “coolant fluid*”[Title/Abstract] OR “industrial fluid*”[Title/Abstract]) AND (“Neoplasms”[MeSH Terms] OR “cancer*”[Title/Abstract] OR “tumor*”[Title/Abstract] OR “neoplasm*”[Title/Abstract] OR “neoplasia*”[Title/Abstract] OR “malignanc*”[Title/Abstract] OR “malignant”[Title/Abstract] OR “benign”[Title/Abstract] OR “carcinoma*”[Title/Abstract] OR “sarcoma*”[Title/Abstract] OR “oncology”[Title/Abstract])).
Scopus	TITLE-ABS-KEY ((metalworking AND fluid*) OR (metal AND working AND fluid*) OR (cutting AND fluid*) OR (mwf) OR (mwfs) OR (lubricant AND fluid*) OR (coolant AND fluid*) OR (industrial AND fluid*)) AND TITLE-ABS-KEY (neoplasm* OR cancer* OR neoplasia* OR malignanc* OR malignant* OR benign* OR carcinoma* OR sarcoma* OR oncology).
Embase	(‘metalworking fluid’/exp OR (metalworking AND (‘fluid’/exp OR fluid)) OR “metalworking fluid*”:ti,ab,kw OR “metal working fluid*”:ti,ab,kw OR “cutting fluid*”:ti,ab,kw OR “metal fluid*”:ti,ab,kw OR “MWF”:ti,ab,kw OR “MWFs”:ti,ab,kw OR “lubricant fluid*”:ti,ab,kw OR “coolant fluid*”:ti,ab,kw OR “industrial fluid*”:ti,ab,kw) AND (‘neoplasm’/exp OR “tumor*”:ti,ab,kw OR cancer*:ti,ab,kw OR “neoplasm*”:ti,ab,kw OR “neoplasia*”:ti,ab,kw OR “malignanc*”:ti,ab,kw OR “malignant”:ti,ab,kw OR “Benign”:ti,ab,kw OR “carcinoma*”:ti,ab,kw OR “sarcoma*”:ti,ab,kw OR “oncology”:ti,ab,kw).
Web of Science	(TS=(“metalworking fluid” OR “metal working fluid” OR (“metalworking” AND “fluid”) OR “cutting fluid” OR “metal fluid” OR “MWF” OR “MWFs” OR “lubricant fluid” OR “coolant fluid” OR “industrial fluid”)) AND (TS=(“cancer” OR “tumor” OR “neoplasm” OR “neoplasia” OR “malignant” OR “malignant” OR “benign” OR “carcinoma” OR “sarcoma” OR “oncology”))

### Inclusion criteria

Studies were included into the meta-analysis if they:

1. Reported the association between exposure to metal-working fluids and incidence of prostate cancer or mortality from prostate cancer.
2. Focused on occupational exposure to metalworking fluids.
3. Observational studies (cohort or case-control) with relevant outcome measures.
4. Published articles with full text available in any language were eligible to be included.

### Exclusion criteria

1. Studies worked on other types of cancer.
2. Studies not related to metalworking fluid exposure.
3. Experimental studies, reviews and case reports.

### Data selection

Two independent reviewers conducted a two-stage screening process. In the first stage, titles and abstracts were screened for relevance based on inclusion and exclusion criteria. In the second stage, full-text articles of potentially relevant studies were retrieved and assessed for eligibility. Disagreements were resolved through discussion or consultation with a third reviewer.

### Data extraction

A standardized data extraction form was developed, including the following information:

- Study characteristics (author, publication year, study design).
- Participant demographics.
- Exposure details (type of metalworking fluid, duration, intensity).
- Standard Mortality Ratio (SMR) and Relative Risk (RR) and associated measures.

Data extraction was performed independently by two reviewers, and any discrepancies were resolved through discussion or consultation with a third reviewer.

### Risk of bias assessment

The risk of bias in individual studies was evaluated using the Joanna Briggs Institute (JBI) Critical Appraisal Checklist for Cohort Studies. This tool assesses studies based on criteria such as selection bias, confounding variables, exposure measurement, and outcome assessment. Each study was independently assessed by two reviewers, and any discrepancies were resolved through discussion or consultation with a third reviewer (35).

### Publication bias

Publication bias, a potential source of systematic error, was assessed using funnel plots and statistical tests. Funnel plots were examined for asymmetry, which may indicate the not presence of publication bias. Additionally, statistical tests such as Egger’s test and Begg’s test were employed to quantitatively assess publication bias.

### Data synthesis

Our study investigated the association between exposure to metalworking fluids and the incidence or mortality related to prostate cancer. RR measures the relationship between exposure to MWF and prostate cancer, while SMR measures the relationship between exposure to MWF and prostate cancer mortality. We employed statistical measures such as Relative Risk (RR) and SMR as indices for our analysis. To synthesize the findings from primary studies, we adopted a methodological approach where, if multiple estimates were available within a category, we applied a fixed-effects model to consolidate the reported results into a singular effect estimate per study. Subsequently, the mean effect size for each study was integrated into the primary analysis. In our primary analysis, studies reporting RR and SMR were treated separately, and we computed overall RR and overall SMR using a fixed-effects model. These overarching effect sizes (SMR and RR) were subsequently integrated into the primary analysis. Under the assumptions of absent heterogeneity and the presence of a genuine underlying effect size, we employed a fixed-effects model to ascertain the final overall effect size, with relevant indices reported accordingly. To gauge between-study heterogeneity, Cochran’s Q and the I² statistic were utilized. Additionally, we employed the Egger test and funnel plot to evaluate potential publication bias. All statistical analyses were conducted using STATA version 17 software. Efforts to adjust for confounding variables in the included studies were variable. Some studies controlled for factors like age and duration of employment, but none comprehensively adjusted for lifestyle factors (e.g., smoking, diet) or comorbidities, which are recognized risk factors for prostate cancer. This represents a limitation of the current analysis and underscores the need for more detailed data collection in future investigations.

## Results

### Literature search

**Figure 1:**
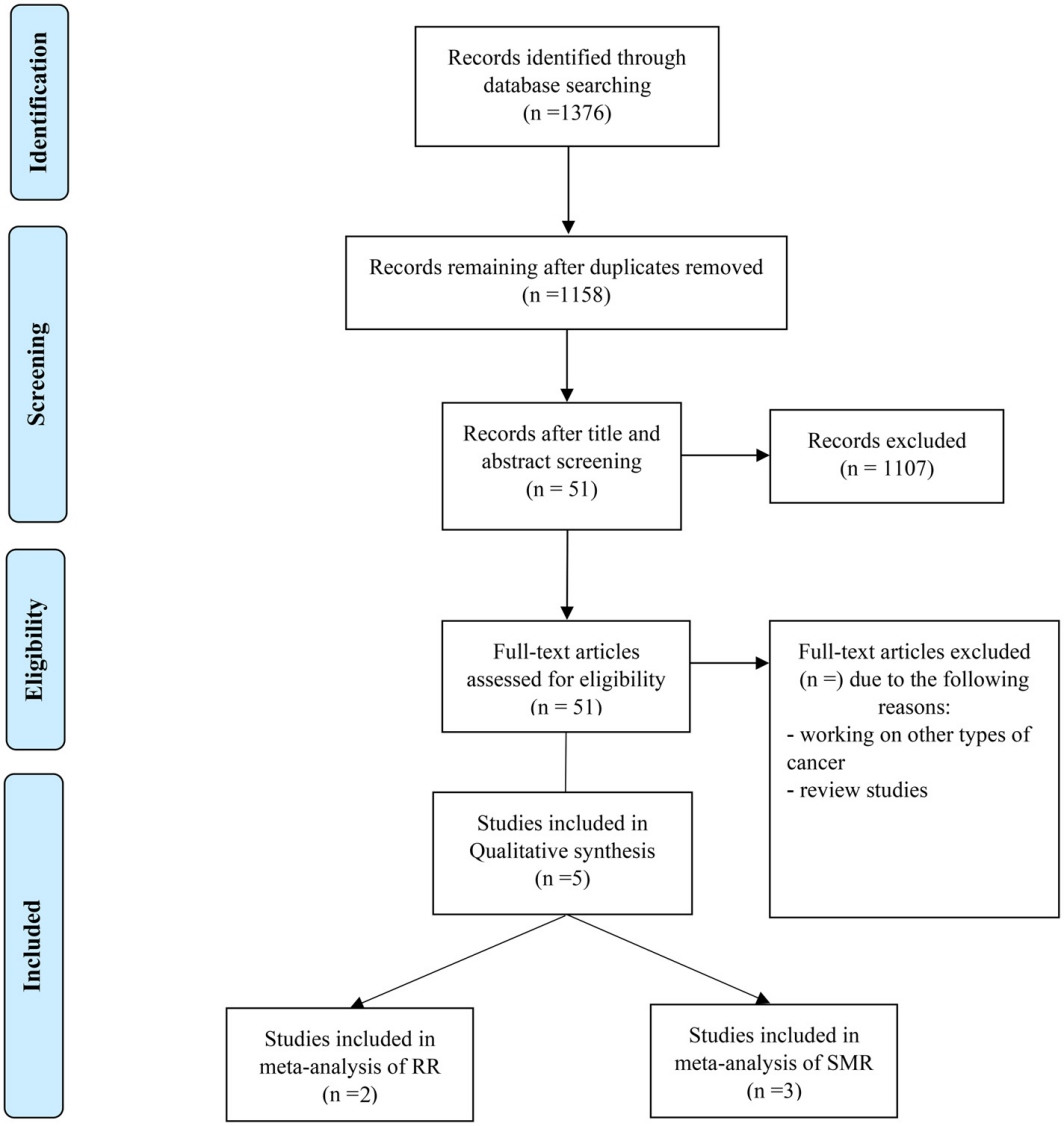
Prisma flowchart indicating the process of including studies in qualitative and quantitative synthesis.

After removal of duplicates, 1376 unique references were screened for inclusion. of those, after exclusion based on title and abstract, 51 papers were obtained in full-text. In total, 5 unique papers meeting the inclusion criteria were used in this meta-analysis. The systematic review included three cohort studies investigating the SMR and two included cohort studies investigating the Relative Risk (RR) in prostate cancer patients with exposure to metalworking fluids.the literature search identified 487 references (**Figure 1**).

### Characteristics of included studies

#### Studies that reported standardized mortality ratio

The studies included in the SMR analysis are summarized in **Table 2**. All studies were cohort designs conducted in the USA, focusing on auto workers or workers in motor vehicle manufacturing, with varying exposure durations to different types of MWFs.

**Table 2:** The characteristics of the included studies according to SMR.

First Author	Year	Country	Study Design	Sample Size	Exposure Duration	Type of MWF	Job	SMR	Lower Limit	Upper Limit
**Tolbert** (36)	1992	USA	cohort	33619	–	Straight Fluids	Auto Worker	1.16	0.91	1.46
						Soluble Fluids	Auto Worker	1.08	0.9	1.28
						Synthetic Fluids	Auto Worker	1.11	0.73	1.63
**Costello** (37)	2020	USA	cohort	38549	–	Synthetic Fluids	Auto Worker	1.3	0.89	1.89
**Delzel** (38)	1993	USA	cohort	223531	13 Years	Not specified	Motor Vehicle Manufacturing	1.24	1.02	1.5
						Not specified	Motor Vehicle Manufacturing	1.36	1.06	1.72

Tolbert (1992) (36) conducted a cohort study involving 33,619 auto workers. The study did not specify the exposure duration but examined exposure to straight, soluble, and synthetic fluids. The SMR for straight fluids was 1.16 (95% CI: 0.91-1.46), for soluble fluids 1.08 (95% CI: 0.90-1.28), and for synthetic fluids 1.11 (95% CI: 0.73-1.63).

Costello (2020) (37) included 38,549 auto workers in a cohort study. Like Tolbert, the exposure duration was not specified, focusing on synthetic fluids with an SMR of 1.3 (95% CI: 0.89-1.89).

Delzell (1993) (38) analyzed a larger cohort of 223,531 workers in motor vehicle manufacturing with an average exposure duration of 13 years. The SMR for this group, with unspecified types of MWFs, was 1.24 (95% CI: 1.02-1.5). For another subset of workers, the SMR was 1.36 (95% CI: 1.06-1.72).

#### Studies that reported relative risk

The studies that reported Relative Risk (RR) are detailed in **Table 3**. These studies comprised a mix of cohort and nested case-control designs, also conducted in the USA, focusing on auto workers exposed to different types of MWFs over various durations.

**Table 3:** The characteristics of the included studies according to RR.

First Author	Year	Country	Study Design	Sample Size	Job	Exposure Duration	Type Of MWF	Relative Risk	Lower Limit	Upper Limit
**Agalliu** (39)	2005	USA	Nested Case-Control	31648	Auto Worker	<25 Years	Straight Fluids	1.117	1.041	1.199
						<25 Years	Soluble Fluids	0.96	0.865	1.065
						>25 Years	Synthetic Fluids	1.072	0.846	1.359
**Eisen** (40)	2001	USA	Cohort	46399	Auto Worker	–	Straight Fluids	0.94	0.83	1.06
							Soluble Fluids	1.13	1.01	1.27
							Synthetic Fluids	1.17	0.91	1.5

Agalliu (2005) (39) conducted a nested case-control study with 31,648 auto workers. For workers exposed to straight fluids for less than 25 years, the RR was 1.117 (95% CI: 1.041-1.199). For those exposed to soluble fluids for less than 25 years, the RR was 0.96 (95% CI: 0.865-1.065), and for synthetic fluids for more than 25 years, the RR was 1.072 (95% CI: 0.846-1.359).

Eisen (2001) (40) conducted a cohort study involving 46,399 auto workers. The study did not specify the exposure duration but reported RRs of 0.94 (95% CI: 0.83-1.06) for straight fluids, 1.13 (95% CI: 1.01-1.27) for soluble fluids, and 1.17 (95% CI: 0.91-1.5) for synthetic fluids.

These studies collectively provide a comprehensive view of the relationship between exposure to various types of metalworking fluids and the incidence or mortality of prostate cancer in different worker populations.

### Meta-analysis results

**Figure 2:**
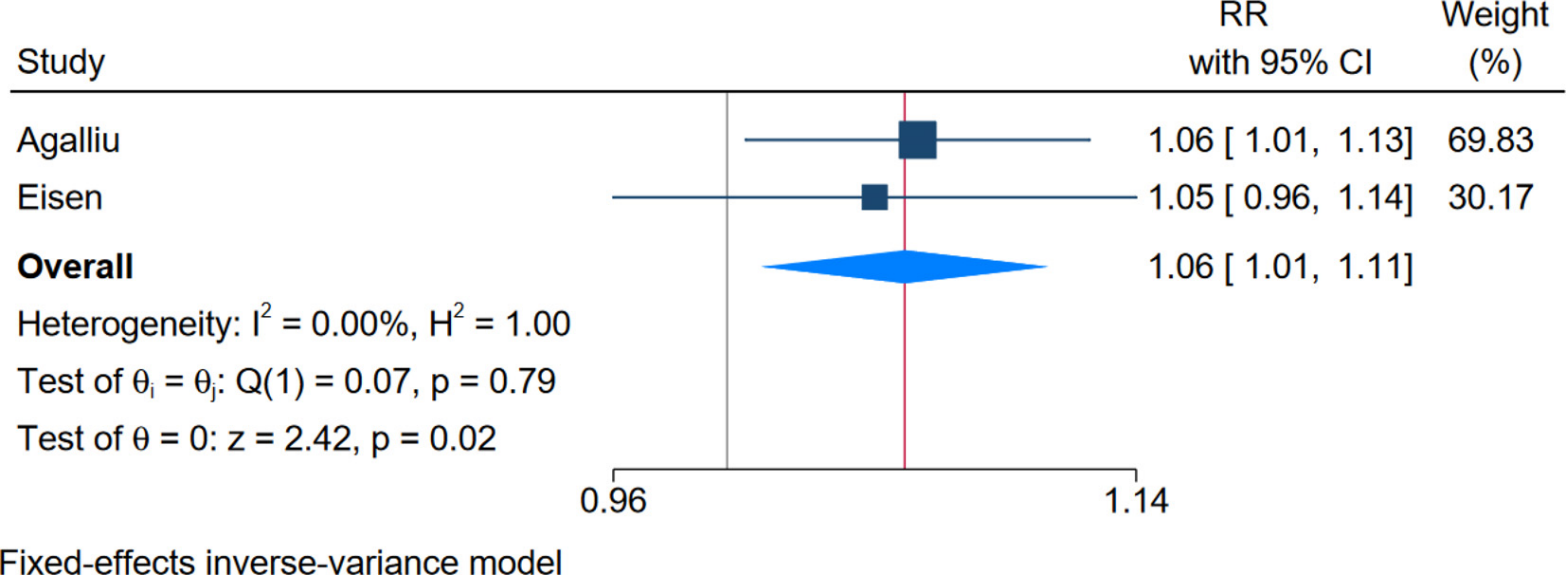
Forest plot of the overall effect size of RR.

**Metal working fluids and incidence of prostate cancer:** The meta-analysis of studies assessing the RR of prostate cancer among workers exposed to metalworking fluids revealed an overall RR of 1.06 (95% CI: 1.01-1.11), indicating a statistically significant 6% increased risk of prostate cancer in the exposed population compared to the non-exposed population. The degree of freedom for this analysis was 1. Heterogeneity analysis showed an I² of 0 and an H² of 1.00, suggesting no significant heterogeneity among the included studies. The Q-test value was 0.07 (p-value = 0.79), further indicating homogeneity. The Z-test value of 2.42 (p-value = 0.02) confirms that the overall RR is statistically significant (**Figure 2**).

**Figure 3:**
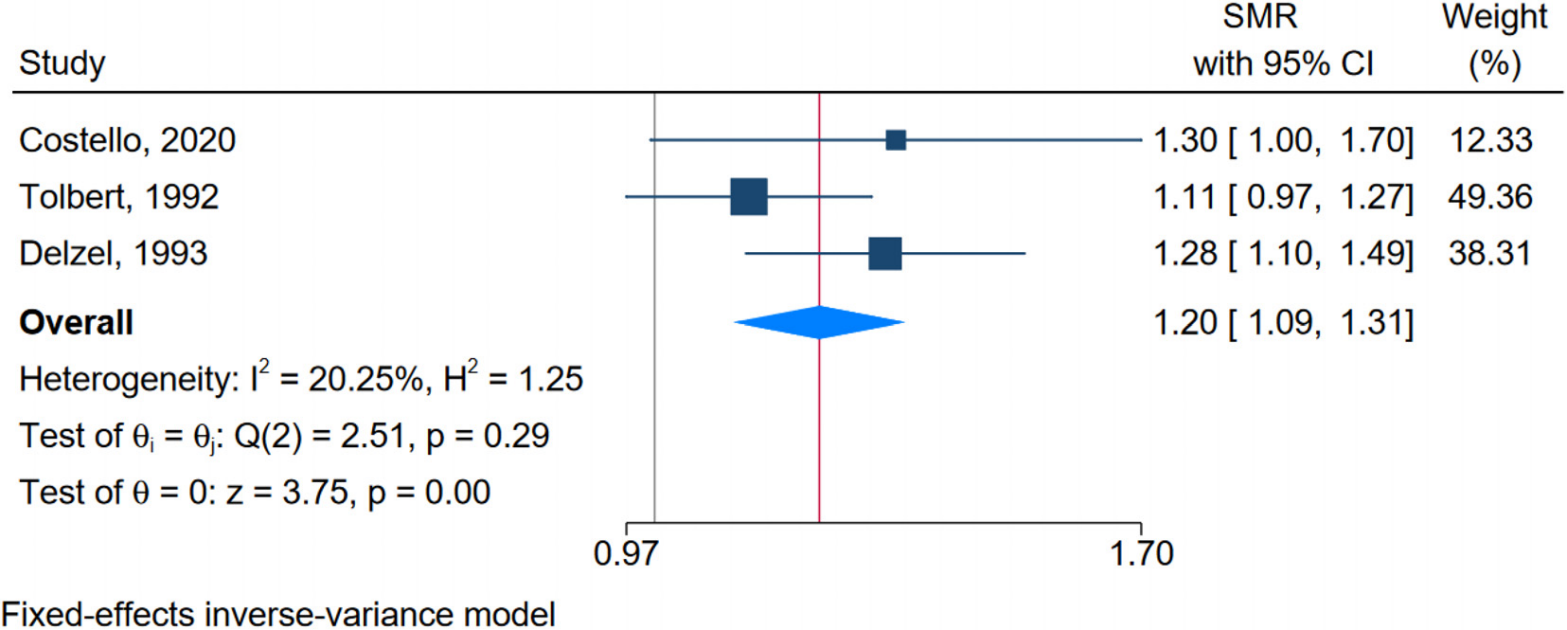
Forest plot of the overall effect size of SMR.

The meta-analysis for the SMR yielded an overall SMR of 1.20 (95% CI: 1.09-1.31), suggesting a 20% increased mortality risk from prostate cancer among workers exposed to MWFs compared to the general population. The degree of freedom for this analysis was 2. The heterogeneity analysis showed an I² of 20.25 and an H² of 1.25, indicating low to moderate heterogeneity. The Q-test value was 2.51 (p-value = 0.29), suggesting that the variation among the study results is not statistically significant. The Z-test value of 3.75 (p-value = 0.00) indicates a highly significant overall SMR (**Figure 3**).

### Publication bias analysis results

**Figure 4:**
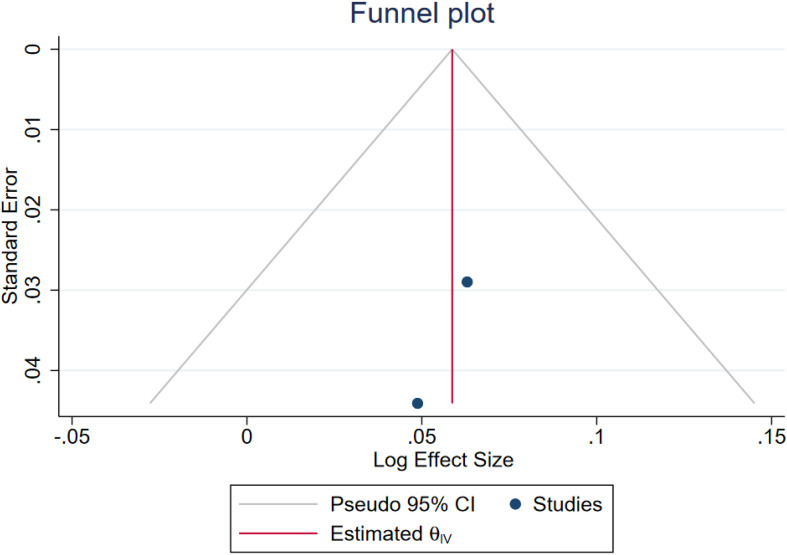
Funnel plot assessing the publication bias among the included RR-reported studies.

**Figure 5:**
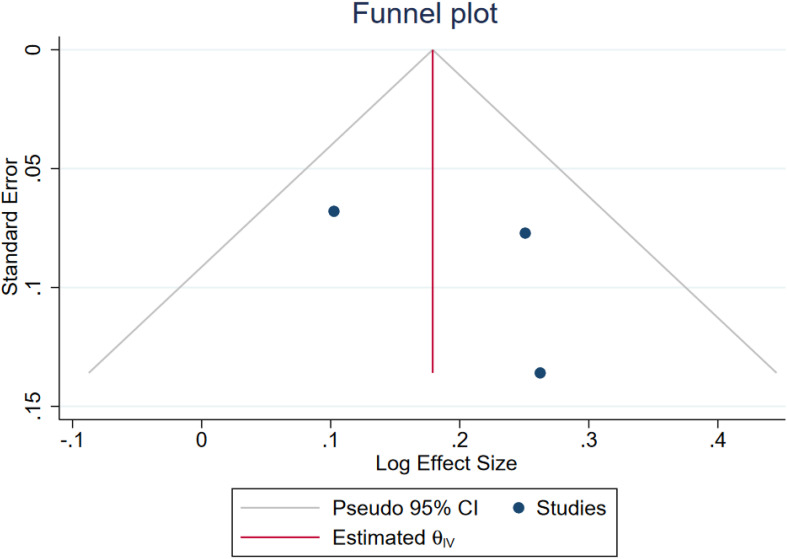
Funnel plot assessing the publication bias among the included SMR-reported studies.

Egger test for small-study effects yielded a beta coefficient of 1.87 with a standard error of 2.512, resulting in a z-score of 0.75 (p = 0.4558), indicating no significant evidence of publication bias. Additionally, Begg’s test for small-study effects showed a Kendall’s score of 1.00 with a standard error of 1.915, resulting in a z-score of 0.00 (p = 1.00), further suggesting no evidence of publication bias. These results are in line with symmetric funnel plots (**Figures 4**, **5**).

## Discussion

While the studies included in this analysis accounted for some confounding variables, such as age and occupational exposure levels, several key potential confounders were not fully addressed. Factors like lifestyle behaviors (e.g., smoking, diet, and physical activity), comorbidities (e.g., diabetes, cardiovascular disease), and concurrent occupational exposures to other potentially carcinogenic agents (e.g., heavy metals, solvents) could influence the observed associations between MWF exposure and prostate cancer risk. Notably, smoking and dietary patterns have been linked to prostate cancer risk in prior studies and may act as significant confounders in occupational cohorts. However, due to data limitations, adjustments for these variables were not consistently performed across all included studies. Future research should prioritize the inclusion of these factors in study designs to enhance the robustness of observed associations. The biological mechanisms linking exposure to MWFs and prostate cancer risk remain incompletely understood. However, toxicological studies have suggested that certain chemical components of MWFs, including nitrosamines, polycyclic aromatic hydrocarbons (PAHs), and biocides, may possess carcinogenic properties. For example, nitrosamines have been shown to induce DNA damage and promote tumor formation in animal models. Additionally, chronic inflammation and oxidative stress resulting from prolonged exposure to these substances could contribute to prostate carcinogenesis. These findings provide a plausible biological rationale for the associations observed in our meta-analysis. Further toxicological and mechanistic studies are needed to elucidate these pathways and strengthen the causal link between MWF exposure and prostate cancer.

MWFs contain a variety of chemicals that may contribute to prostate carcinogenesis through distinct biological pathways. Contaminants such as nitrosamines, polycyclic aromatic hydrocarbons (PAHs), and biocides have been implicated in DNA damage, oxidative stress, and hormonal disruptions. Nitrosamines, for instance, are potent carcinogens known to induce mutations and promote tumor formation in animal models. PAHs, another class of compounds frequently found in MWFs, have been linked to oxidative DNA damage and the activation of oncogenic pathways. Hormonal disruptions may occur through exposure to endocrine-disrupting chemicals in synthetic MWFs, which could contribute to an altered hormonal milieu that predisposes individuals to prostate cancer. These mechanisms underscore the need for further toxicological and molecular studies to confirm these associations and strengthen causality.

In addition to identifying the genes involved in the hereditary form of prostate cancer, many studies have also investigated the mutations that occur in the acquired form. Therefore, detailed analysis of prostate cancer epidemiology and assessment of risk factors will help to understand the relationship between genetic mutations and the role of the environment and occupation in creating these mutations and preventing tumor progression (41, 42). One of the possible environmental factors is metalworking fluids. Although there is provocative evidence based on MWF contaminants that can cause tumors in laboratory animals (43) and significant levels of occupational exposure to MWF, but few studies have been conducted on the risk of prostate cancer. Prostate cancer is the second most common malignant cancer (after lung cancer) in men worldwide (44). International mortality for prostate cancer varies considerably around the world. In 2018, the highest mortality rates were recorded in Central America, followed by Australia and New Zealand and Western Europe. The lowest rate is reported in Asian and North African countries (45).The cause of prostate cancer has been investigated in numerous studies and remains largely unknown. Proven risk factors for prostate cancer include: increasing age, ethnicity, genetic factors and family history (46, 47). Other possible factors associated with prostate cancer include diet, obesity and lack of exercise, inflammation, hyperglycemia, infections, and environmental exposure to chemicals or ionizing radiation (48, 49).

The results of the present study demonstrated a statistically significant increased mortality and relative risk of prostate cancer associated with exposure to metalworking fluids. After reviewing articles found between 1990 and 2023, five articles related to metalworking fluids and prostate cancer remained and were considered for meta-analysis. Four cases were cohort studies and one case was nested case-controlstudy related to the exposure of automotive workers to metalworking fluids. A meta-analysis with fixed effects was performed for the mentioned studies. The overall effect size of SMR and RR in prostate cancer mortality among metalworking fluid exposed subjects was obtained 1.20 (95% CI: 1.09 to 1.31) and 1.06 (95% CI: 1.01 to 1.11), respectively. In the study, Lee et al. investigated the occurrence of cancer caused by metalworking fluids in metal workers. The SMR values reported in the mentioned study in two separate factories were 1.02 and 1.03 (50). Costello et al., also confirmed the relationship between exposure to metalworking fluids and mortality from cancers of the esophagus, stomach, intestine, rectum, bladder, liver, pancreas, larynx, lung, skin, prostate, brain, breast, and also Leukemia (39). The results of these studies are consistent with the present study.

Nowadays, with the development of production of metalworking fluids, chemical anti-corrosion inhibitors such as amine are replaced by borate components in order to reduce the formation of nitrosamines. To provide biostability along with corrosion inhibition, boron compounds containing water-soluble inhibitors, including amine borates, commonly referred to as borate esters and amine carboxylate derivatives, have been added to the formulation of metalworking fluids (144). Although the production of molecules with the ability to create nitrosamines has recently decreased in metalworking fluids containing boron compounds, but it seems that this replacement is not very suitable and after using these metalworking fluids in the industry, compounds with higher genotoxicity have been created (51). However, no study in this regard was found among the searches of the present study. One of the reasons for the non-significance of different metalworking fluids with prostate cancer can be the small sample size for each metalworking fluid.

The variability in the composition of MWFs—straight oils, soluble oils, and synthetic fluids—poses a challenge in disentangling the specific risks associated with each type. Studies in our meta-analysis reported varying effect sizes depending on the fluid type; however, the small number of studies and lack of detailed exposure data limited the ability to perform a subgroup analysis by MWF type. For instance, exposure to straight fluids was associated with higher SMR and RR values in some studies, whereas synthetic fluids showed inconsistent results. This highlights a critical limitation in the current literature. Future studies should aim to disaggregate risks by MWF type, considering differences in chemical composition and their respective carcinogenic potentials.

Although heterogeneity among the included studies was low to moderate, the inability to fully control for confounding factors such as smoking, alcohol consumption, dietary habits, and genetic predisposition remains a significant limitation. Smoking and diet, in particular, are well-established risk factors for prostate cancer and may have influenced the observed associations. Additionally, occupational co-exposures, such as exposure to heavy metals or solvents, may confound the relationship between MWF exposure and prostate cancer risk. Addressing these factors requires more robust study designs and comprehensive data collection in future research.

Recently, there have been significant changes in the composition of metalworking fluids, but currently, newer chemicals are consistently used with little information on health and safety hazards. Therefore, considering the existence of few studies in relation to metalworking fluids and prostate cancer, it seems that there is a need for more and newer studies in relation to metalworking fluids with new compounds.

## Data Availability

The original contributions presented in the study are included in the article/supplementary material. Further inquiries can be directed to the corresponding authors.
